# Chromatic Contrast Sensitivity Functions and Colour Discrimination in Smoker Patients

**DOI:** 10.3390/ijerph19126991

**Published:** 2022-06-07

**Authors:** Mari Carmen García-Domene, María Josefa Luque-Cobija, Dolores de Fez, María Amparo Díez-Ajenjo

**Affiliations:** 1 Optics, Optometrý and Visual Sciences, University of Valencia, 46100 Burjassot, Valencia, Spain; m.carmen.garcia-domene@uv.es (M.C.G.-D.); maria.j.luque@uv.es (M.J.L.-C.); 2 Optics, Pharmacology and Anatomy Department, University of Alicante, 03690 Sant Vicent del Raspeig, Alicante, Spain; dolores.fez@ua.es

**Keywords:** contrast sensitivity, Farnsworth–Munsell, smoker, total error score, colour

## Abstract

In this study, effects of smoking on colour vision with the Farnsworth–Munsell 100 Hue test (FM100h) and achromatic (A), red-green (RG), and blue-yellow (BY) contrast sensitivity functions were evaluated. In total, 50 non-smoker controls and 25 smokers, divided into two groups (group 1, less than 10 cigarettes per day, with 15 patients, and group 2, >10 cigarettes per day, with 10 patients) took part in the experiments. Best-corrected visual acuity (BCVA), FM100h, and A, RG, and BY contrast sensitivity functions were measured. Total and partial RG and BY error scores (*TES* and *PTES*) and colour axis index (*CA*) were used in the analysis. No differences between smoker and non-smoker groups were found in BCVA, *CA* and A and BY contrast sensitivity, but *TES* and *PTES* values and RG contrast sensitivity at 1 cpd were statistically different. Differences between smoker groups were not significant. Error scores in smokers were positively correlated with the number of cigarettes smoked per day, and in BY also with age. Tobacco caused discrimination losses in both chromatic mechanisms but affected the red-green pathway more than the blue-yellow, and therefore, a partial RG score of FM100h test seems to be a good predictor of smoker colour deficiencies.

## 1. Introduction

The compounds in cigarettes and cigarette smoke, which comprise more than 4500 toxic substances and heavy metals (nicotine, nitrosamines, benzene, carbon monoxide, etc.) [1,2,3,4], are associated with carcinogenic, toxic, and mutagenic effects on health [5]. In the visual system, smoking is associated with early onset or the development of ocular pathologies such as age macular degeneration [1,2,3,4], cataracts [1,3], thyroid-associated ophthalmopathy [1,2,3], anterior ischemic optic neuropathy [3], toxic amblyopia (alcohol-tobacco amblyopia) [3,4], hypertensive retinopathy [3], uveitis [1], ocular surface damage [1,4,6,7,8,9,10], and macular pigment losses [2]. Smokers suffer from functional losses affecting visual pathways, caused by retinal hypoxia and carbon monoxide poisoning [11]. These losses produce reductions in chromatic sensitivity in the mesopic range [12] and central or paracentral scotomas in standard achromatic perimetry [13,14].

Dose-dependent colour vision alterations, as assessed by colour ordering tests, have been observed in the central retina. Measurements with the desaturated Roth 28-Hue test reveal differences in error scores between non-smokers, smokers of less than 20 cigarettes a day and smokers of more than 20 cig/day [15], and smokers in the 20–34 age range who had been smoking at least 20 cigarettes per day, for at least a year, had statistically less sensitivity to red-green differences in the D-15 test [16]. On the other hand, non-selective chromatic discrimination losses in smokers have also been reported [17].

Previous studies on red-on-white [13] or blue-yellow [14,18] (SWAP) perimetry with smokers classified according to the number of cigarettes smoked per day, found foveal sensitivity losses in moderate smokers (maximum 20 cig/day) [14], and paracentral scotomas in chronic smokers (minimum 20 cig/day) [13]. Losses in the blue-yellow mechanism, however, are not significant in young moderate smokers, showing a possible effect of age [19].

Our aim is to detect and compare possible losses in the achromatic (Ac), red-green (RG), and blue-yellow (BY) mechanisms of smokers, and their dependence on age and the number of daily smoked cigarettes. To compare the two chromatic mechanisms, red-green and blue-yellow partial scores of the Farnsworth–Munsell 100 hue test (FM100h) are used. However, when considering the achromatic mechanism, the assessment of the relative losses in the different visual pathways would be easier if the same psychophysical task were used to evaluate all mechanisms, although this strategy is not widespread in the literature. In this study, the common task is a contrast sensitivity measurement, with stimuli chosen to favour the responses of each mechanism.

## 2. Materials and Methods

This study adhered to the tenets of the Declaration of Helsinki for Research Involving Human Subjects and was approved by the University of Valencia Institutional Review Board (H20190404172821). All the patients involved in this study signed an informed consent form to participate in the study.

In this study, we compared a smoker and a non-smoker control group in the 20–60 age range. General exclusion criteria were retinopathies or any ocular or systemic pathologies that could affect the results, previous ocular surgery, ocular treatments during the month prior to the commencement of the study, and medication that could produce somnolence (for instance, antihistamines) or a history of drug addiction or alcoholism. Patients with congenital colour vision deficiency were excluded.

Non-smokers were required not to have smoked more than 100 cigarettes sporadically throughout life. Subjects living with smokers were discarded. Selected smokers had been smoking cigarettes for at least 10 years and were separated into two subgroups. Group 1 includes smokers consuming between 10 and 20 cigarettes per day (moderate consumers) and Group 2 includes smokers consuming more than 20 cigarettes per day (severe consumers). No distinction was made between cigarettes and hand-rolling tobacco because both are equally harmful [20].

All the observers underwent an exhaustive ophthalmological exam, including a questionnaire about health habits, full anamnesis, slit-lamp exam (SL-2F Topcon), measurement of the best-corrected visual acuity (BCVA) with an EDTRS chart, optical compensation, and determination of the intraocular pressure (IOP) with a non-contact tonometer (TRK-1P Topcon). Patients wore the optical compensation determined throughout the experiments. Lenses with blue-blocking filters were avoided. Only the right eye was included in the study, and this was chosen at random for the whole sample. In patients with problems in this eye (f.i. amblyopia), the left eye was chosen.

**Figure 1:**
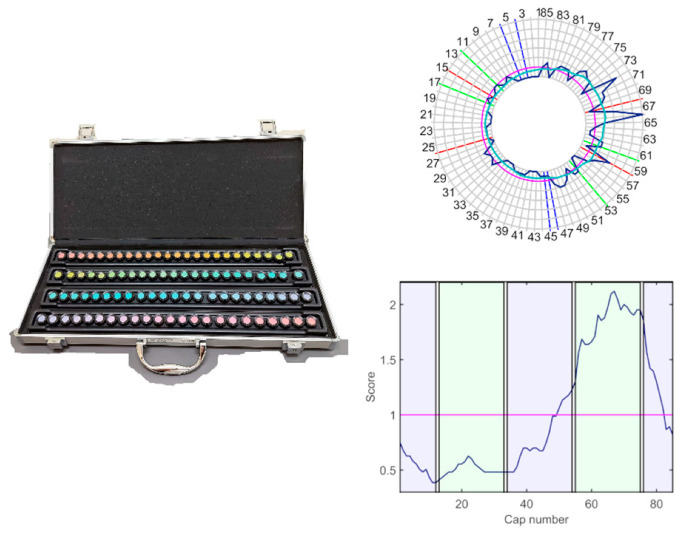
(**Left**) the Farnsworth–Munsell 100 hue test. (**Right**) example of the standard result diagram, showing the original Farnsworth plot (**top**, blue line), its low-pass-filtered version (**top**, cyan line), and the Dain–Birch (DB) plot (**bottom**). The coloured sections of the DB plot represent which caps are sorted according to RG (green) and BY (bluish) mechanisms. The magenta line is the patient’s normalised mean score. The example shows general discrimination losses that are worse for the red-green mechanism.

The function of the chromatic and achromatic mechanisms was evaluated by means of the FM100h test and the contrast sensitivity function (CSF), with only the light provided by the test stimuli, in an otherwise darkened room (less than 1 lux on the plane of the test). The FM100h (Figure 1) was administered under D65 illumination provided by a Sol-Source Gretag-Macbeth daylight simulator lamp, providing 950 luxes on the plane of the samples. The FM100h is an ordering test designed to evaluate the chromatic mechanisms and is composed of 85 chips with constant value and chroma that cover all the visual hues described by the Munsell colour system. The chips are distributed into four boxes, with two fixed samples in their extremes. Patients must order the samples by choosing the chip more similar to the last one placed in the box.

Chromatic and achromatic contrast sensitivity functions were measured along the achromatic (A), red-green (RG), and blue-yellow (BY) cardinal directions of the Derrington–Krauskopf and Lennie colour space [21] using sinusoidal stimuli on a 45 cd/m^2^ achromatic background, (*x_CIE_* = 0.2915, *y_CIE_* = 0.3147). The spatiotemporal stimulus profile is defined by Equation (1) as follows:

(1) $$\left(\begin{array}{c} \Delta A\left(x,y,t\right)\\ \Delta RG\left(x,y,t\right)\\ \Delta BY\left(x,y,t\right) \end{array}\right)=\left(\begin{array}{c} \Delta A_{s}\\ \Delta RG_{s}\\ \Delta BY_{s} \end{array}\right)\cdot cos\left(2\pi f_{x}\right)\cdot h\left(t\right)\cdot rect\left(\frac{x}{a},\frac{y}{a}\right)$$

where vector (Δ*A_S_* Δ*RG_S_* Δ*BY_S_*) defines the modulation direction in the colour space and has always two zero components. The grating’s spatial frequency is *f_x_*, the stimulus size, a, is 5°, and *h*(*t*), defined by Equation (2), smooths the temporal profile of the stimulus to avoid temporal transients.

(2) $$h\left(t\right)=\{\begin{array}{cc} \exp\left\{-\frac{{\left(t-t_{0}\right)}^{2}}{2\sigma_{t}^{2}}\right\} & if\;\;0\leq t\leq t_{0}\\ 1 & if\;\;t_{0}<t\leq T_{s}-t_{0}\\ \exp\left\{-\frac{{\left(t-T_{s}+t_{0}\right)}^{2}}{2\sigma_{t}^{2}}\right\} & if\;\;T_{s}-t_{0}<t\leq T_{s} \end{array}$$

The maximum stimulus duration, *T_s_*, was equal to 1 s. Parameters *t*_0_ = 100 ms and *σ_t_* = 100/3 ms, were chosen to ensure zero amplitude at *t* = 0 and *t = T_s_*. The spatial frequencies used were 1, 2, 4, 8, and 16 cpd.

**Figure 2:**
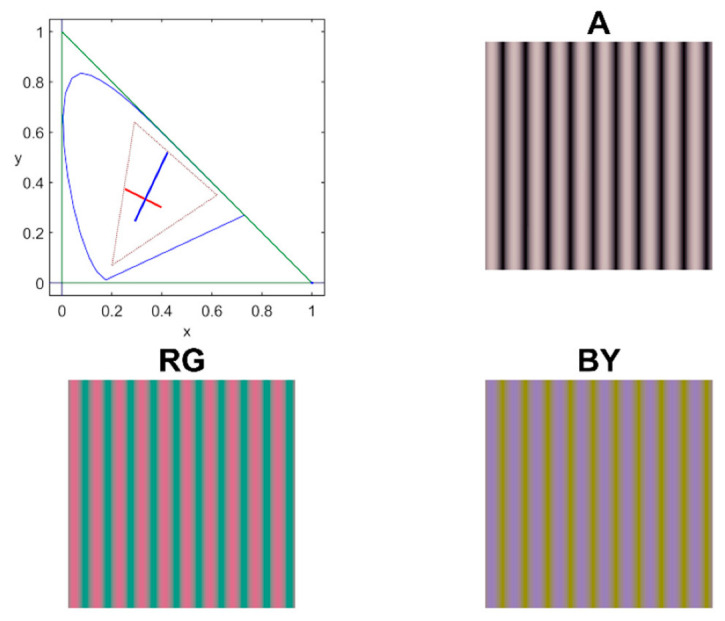
Example of the achromatic (**A**), red-green (**RG**), and blue-yellow (**BY**) sinusoidal patterns used in the CSF measurement. The chromaticity diagram shows the colour palette for the RG (red line) and BY (blue line) with maximum amplitude.

Stimuli were displayed on a 17″ CRT monitor, colourimetrically characterised and gamma-corrected, driven by a Bits++ video controller of 12 bits (Cambridge Research Systems) with the patients placed at 50 cm from the screen, wearing their distance refraction plus a +2D addition, to minimise accommodation. Amplitude threshold, *R_thres_*, for a given frequency was determined using a variant of the staircase procedure, described in detail elsewhere [22]. Contrast sensitivity, *S*, was computed in dB (Equation (3)) as follows:

(3) $$S=Log_{10}\frac{{\Delta R}_{\max}}{{\Delta R}_{\mathrm{thres}}}$$

where Δ*R_max_* is the maximum amplitude for the considered cardinal direction that can be generated by the device. Figure 2 shows an example of the colour palette and the spatial pattern of the stimuli.

### Data and Statistical Analysis

FM100h results were plotted and low-pass-filtered (Figure 1) using Dain and Birch’s (DB) criterion, to determine the regions of selective discrimination loss [23]. To this end, the DB score of the *i*th-cap, $\varepsilon_{DB,i}$, was computed by averaging and normalising the Farnsworth scores in their neighbourhood, as indicated in Equation (4).

(4) $$\varepsilon_{DB,i}=\frac{\sum_{j=i-10}^{i+1}\varepsilon_{F,j}}{\sum_{j=1}^{85}\varepsilon_{F,j,}}$$

where the Farnsworth scores $\varepsilon_{F,j}$, are the sum of the distances between a cap and its two contiguous neighbours. Total error scores (*TES*) [24] were computed using a MATLAB-based program developed by researchers of the Universities of Valencia and Alicante [25]. *TES* scores were computed by summing the error scores, $\varepsilon_{F,j}$, for every cap. To determine the contribution of the red-green and blue-yellow mechanisms to the total error, total partial error scores (*PTES*) were computed for the red-green axis as the sum of errors of caps 13–33 and 55–75, and for the blue-yellow axis as the sum of errors of caps 1–12, 34–54, and 76–85 [23]. The colour axis index (*CA*) [26] was also calculated (Equation (5)).

(5) $$CA=\sqrt{PTES_{BY}}-\sqrt{PTES_{RG}}$$

Statistical analysis was carried out with IBM SPSS Statistics 20 (IBM Corp., Armonk, NY, USA). The normality of the distributions was checked separately for the smoker and non-smoker groups with chi-square or Kolmogorov–Smirnov tests. Due to the non-normal distribution of most of the variables, non-parametric tests were used. To compare results among groups, the H Kruskal–Wallis test with Bonferroni’s criterion for multiple comparisons was used. Correlations among parameters were analysed through Spearman’s rho. Principal components analysis of the perceptual data was carried out, and the dependence on age and amount of smoked cigarettes was explored using linear models. Finally, diagnostic power was evaluated by means of receiver operating characteristics (ROC) curves.

## 3. Results

Data analysis was carried out over a total of 75 observers. The distribution in groups and descriptive parameters are shown at Table 1. The groups did not significantly differ in age or BCVA (*H* = 3.119 *p* = 0.210; *H* = 1.153 *p* = 0.562). Data in Table 1 show a trend for higher *TES* and RG and BY *PTES* for the smokers’ groups in comparison with the control group. These differences in FM100h scores were statistically significant for *TES* (*H* = 24.121, *p* = 0.000), *PTESRG* (*H* = 22.791, *p* = 0.000) and *PTESBY* (*H* = 19.787, *p* = 0.000). Group 0 and 1 were statistically different in *TES* (*H*_0–1_ = −16.685, *p* = 0.023) and *PTESRG* (*H*_0–1_ = −18.084, *p* = 0.011) but not for *PTESBY* (*H*_0–1_ = −14.058 *p* = 0.070). Groups 0 and 2 were significantly different in *TES* (*H*_0–2_ = −33.752, *p* = 0.000), *PTESRG* (*H*_0–2_ = −31.317, *p* = 0.000), and *PTESBY* (*H*_0–2_ = −30.691, *p* < 0.001). There were not statistically significant differences between groups 1 and 2 in *TES* (*H*_1–2_ = −17.067, *p* = 0.146), *PTESRG* (*H*_1–2_ = −13.233, *p* = 0.364), or *PTESBY* (*H*_1–2_ = −16.633 *p* = 0.154).

If subjects are classified as normal (CN) or colour-deficient (CD) using the 95th percentile for *TES* for their age group [24], all controls are within normal limits; however, in this study, 40% of subjects in group 1 and 60% of subjects in group 2 were CD.

A comparison of the partial scores (*PTESRG* and *PTESBY*) between group 0 and groups 1 and 2 showed values of 44% and 62% in *PTESRG* and 29% and 55% for *PTESBY*, respectively. This suggests damage in both the RG and BY mechanisms, and a trend for worse values in the group that consumed more cigarettes (group 2). The *CA* index did not reveal a colour loss axis, but the smokers’ range was displaced towards lower values than in the control group, suggesting worse red-green discrimination in smokers, although the differences between groups were not statistically significant for this parameter.

**Figure 3:**
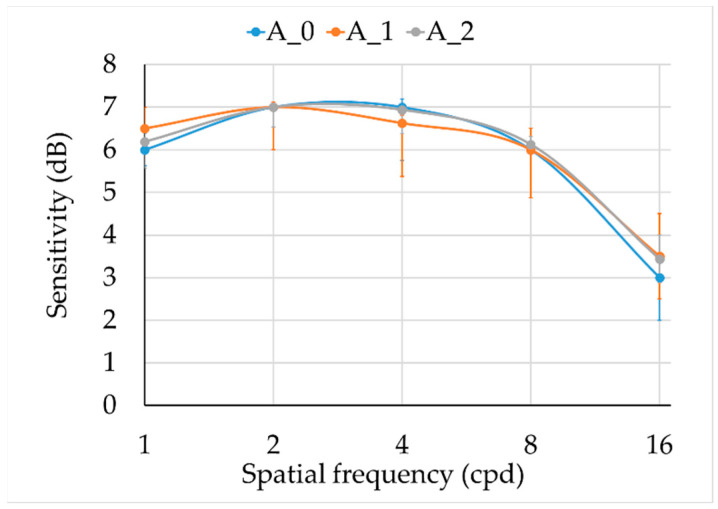
Achromatic monocular CSF. Median and interquartile range for three groups: A_0 = controls (blue), A_1 = moderated smokers (orange), and A_2 = severe smokers (grey).

**Figure 4:**
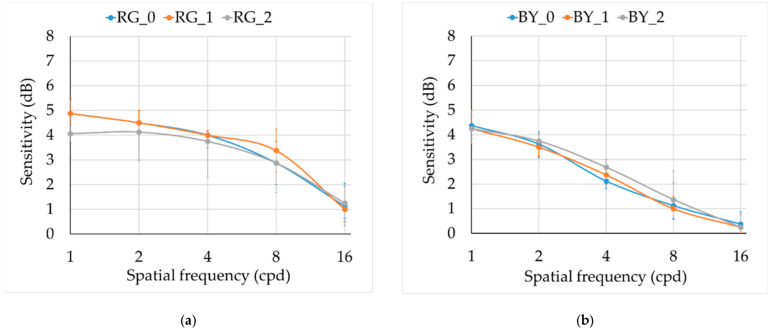
Chromatic monocular CSFs. Median and interquartile range for three groups: 0 = control (blue), 1 = moderated smokers (orange), and 2 = severe smokers (grey): (**a**) RG mechanism, (**b**) BY mechanism.

Results for the achromatic and chromatic CSFs are shown in Figure 3 and Figure 4. There were no statistically significant differences between groups in the achromatic CSF (Figure 3, Table 2), but larger dispersion was observed in smokers.

The medians for the two chromatic CSFs (Figure 4) for the three groups were similar, but the interquartile range was higher for smokers’ groups. In the RG mechanism, CS at 1 cpd was significantly lower for group 2 in comparison with controls (*H_0–2_* = 18.443, *p* = 0.040, with medians 4.06 dB and 4.88 dB for groups 2 and 0, respectively). We did not find statistical differences for the rest of the frequencies (see statistics in Table 2).

**Figure 5:**
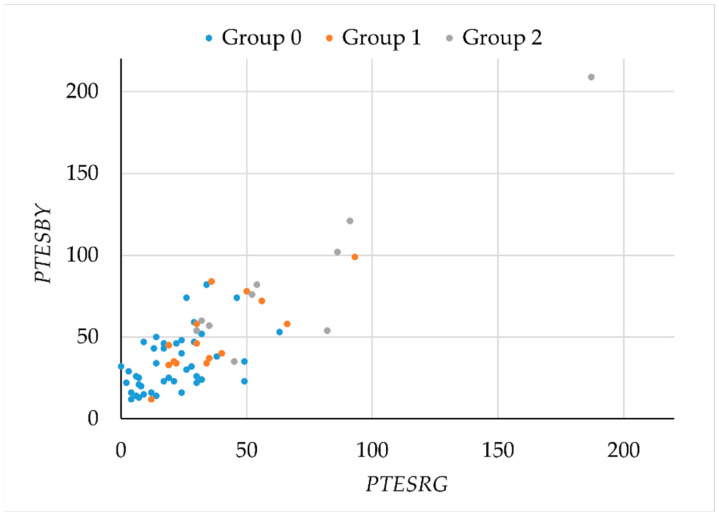
*PTESRG* vs. *PTESBY* monocular results for three groups: 0 = control (blue), 1 = moderated smokers (orange), and 2 = severe smokers (grey).

To study possible losses in the RG and BY chromatic mechanisms, as assessed by the FM100h test, we plotted *PTESBY* versus *PTESRG* (Figure 5). Although the plot suggests a possible separation between smokers and non-smokers, the overlap between the different groups is large. Given the marked orientation of the plot, principal component analysis was used to search for a parameter with better segmentation capabilities.

**Figure 6:**
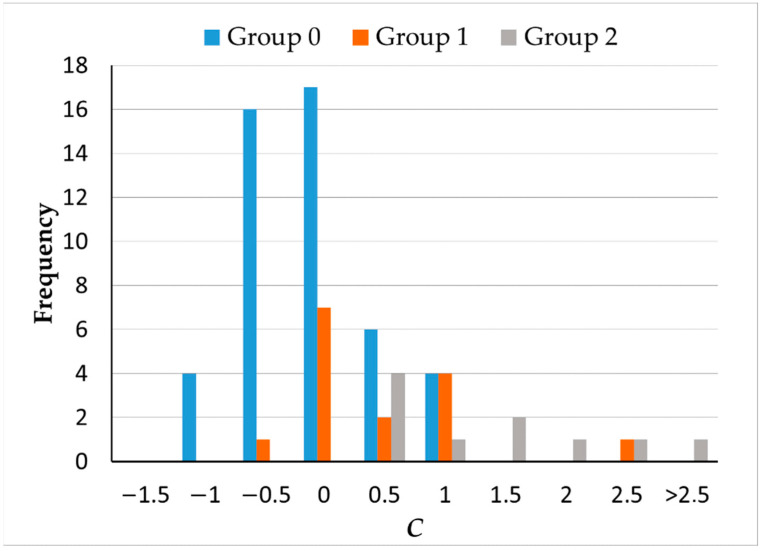
Distributions of principal component *C* for three groups: 0 = controls (blue), 1 = moderated smokers (orange), and 2 = severe smokers (grey).

A single component, *C* = 0.351 *TES* + 0.337 *PTESRG* + 0.338 *PTESB*, explained 95% of the variance. The scores for this component by group are described in Figure 6. Differences between groups were statistically significant (*H* = 23.782, *p* < 0.001). Scores were significantly lower for controls and groups 1 and 2 (*H*_0–1_ = −16.432, *p* = 0.024; *H*_0–2_ = −33.182, *p* < 0.001), but there were no differences between the two groups of smokers (*H*_1–2_ = −16.75; *p* = 0.150).

We analysed the correlations between predictors age and number of consumed cigarettes and output variables (FM100h scores and 1 cpd CSF for RG). For this analysis, we merged groups 1 and 2. Age was correlated with *TES* (*rho* = 0.469, *p* = 0.001), *PTESBY* (*rho* = 0.566 *p* < 0.001) and *C* (*rho* = 0.417 *p* = 0.004) for non-smokers, with higher values for older subjects, as expected, but not for *PTESRG* (*rho* = 0.211 *p* = 0.154), nor for 1 cpd CSF for RG (*rho* = −0.225 *p* = 0.119). For smokers, age was not correlated with any other parameter (*p* > 0.05 in all cases), but the number of consumed cigarettes was positively correlated with *TES* (*rho* = 0.582, *p* = 0.002), *PTESRG* (*rho* = 0.549, *p* = 0.005), *PTESBY* (*rho* = 0.589, *p* = 0.002), and component *C* (*rho* = 0.582, *p* = 0.002), although not for 1 cpd CSF for RG (*rho* = −0.074 *p* = 0.726). Considering these results, a linear regression model was used to determine the influence age and number of smoked cigarettes on perceptual parameters. The results of this analysis are shown at Table 3. All of the FM100h parameters significantly depended on the number of smoked cigarettes, while only *PTESBY* also depended on age.

**Figure 7:**
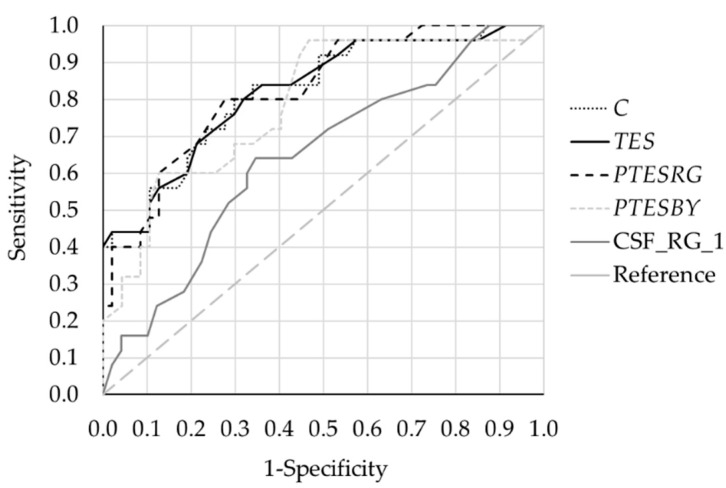
ROC curves for different parameters: FM100h scores (*TES*, *PTESRG*, *PTESBY*, computed principal component *C*), and 1 cpd contrast sensitivity for the red-green mechanism (CSF_RG_1). AUC, area under curve; SENS, sensitivity; SPEC, specificity; CUT_OFF_, cutoff value of the curve.

Finally, to analyse the diagnostic power of the different perceptual parameters, we calculated the receiver operating characteristics (ROC) curves. We compared group 0 (negative) with groups 1 and 2 together (positive), and the results are described in Figure 7 and Table 4. The data extracted from the curves are the area under the curve, specificity, and cutoff value for 80% of sensitivity and sensitivity and cutoff value for 80% specificity. If we consider these data, the best perceptual parameter is *PTESRG*, since it had the best specificity and sensibility, with a cutoff value between 29.5 and 32, although component *C* and *TES* had similar results.

## 4. Discussion

In this paper, we studied the effect of tobacco in moderate and severe smokers on the performance of the achromatic, red-green, and blue-yellow mechanism using the same kind of perceptual task—the measurement of monocular contrast sensitivity functions. The two chromatic mechanisms were also compared by means of the Farnsworth–Munsell 100 Hue test. Although the study is limited by sample size and by the fact that group sizes are dissimilar, some interesting trends emerged. Though both techniques revealed functional damage in smokers, they were not equally sensitive. In CSF measurements, the only significant difference appeared in the low-frequency range in the RG mechanism. This result differs from the reported losses in smokers’ achromatic contrast sensitivity function [15,27]. On the other hand, the FM100h test showed general sensitivity losses in both chromatic mechanisms, which could be slightly worse for RG, although no clear axis of colour discrimination loss was found.

The different sensitivity of CSF and FM100h to separate controls from smokers could just be a consequence of the larger variability in the responses of subjects to the grating detection task. However, it has been shown [28] that a colour-sequencing task activated more colour-selective areas than passive viewing or colour-discrimination tasks such as grating detection. Given that our patients did not present retinal damage detectable by fundus exploration, this could suggest damage due to tobacco in cortical sites. Nevertheless, simple retinal damage has been hypothesised as a likely cause for colour discrimination losses. Smoking has been shown to cause optic neuropathy by damage to the optical nerve. In tobacco optic neuropathy, the symptoms include chromatic discrimination losses that are larger in the red-green direction [29]. It has been hypothesised that these changes in colour vision are caused by the accumulation of toxic substances in blood that affect the retinal pigment epithelium in the retina. This would affect the three-cone types, explaining increments in *TES* [15]. Fletcher and Voke have also suggested cigarettes and carbon monoxide as causes of discrimination losses in the red-green mechanism [30].

The fact remains, however, that the number of daily smoked cigarettes was significantly related to the magnitude of discrimination loss, but again only with the FM100h. Retinal damage could also be expected to affect the contrast sensitivity function, but only low-frequency sensitivity in the RG mechanism reflected such a loss. Though smokers showed worse results than non-smokers in different parameters, our sample was too small to reveal significant differences between moderate and heavier smokers, as occurred with the FM100h results reported by Arda et al. (2010) [31], although they found a significant correlation between *TES* and the number of smoked cigarettes per day. We found a similar effect in *TES*, *PTEST*, and the first-principal component *C*.

Previous literature reports damage in the achromatic, red-green, or blue-yellow [11,12,13,14,15,16,17,18,19] mechanisms, but comparisons between mechanisms are scarce. The published data show variability: although global damage is reported, results are not always consistent with a selective loss pattern, and when this selective loss is found, there is no agreement on which is the more affected mechanism.

The detection of damage may depend both on the task that is asked from the observer and on the parameter used to evaluate performance. In the FM100h test, for instance, the colour axis index did not reveal selective losses even when *TES* signalled damage in the chromatic mechanisms [32], in contrast to *PTES*, as in our case.

However, other studies found selectively larger damage for the BY mechanism, though this mechanism could be more resilient than RG in the long term [33]. Some authors measured the discrimination threshold from white along the L, M, and S cone-isolating directions using the Cambridge Colour Test [17,33]. Monteiro de Paiva Fernandes et al. found selectively worse damage along the red-green directions in deprived smokers and showed a general threshold elevation along the protan, deutan, and tritan directions in smokers. Although the plots suggest greater threshold relative increments along the tritan direction, according to the authors, effect size analysis confirmed that the largest discrimination losses in smokers appeared along the L and M isolating directions. Results with deprived smokers suggest that damage in the red-green mechanism would be of longer duration [33].

## 5. Conclusions

Based on the CSF results, the only significant difference appeared in the low-frequency range in the RG mechanism, whereas the FM100h test showed general sensitivity losses in both chromatic mechanisms, which could be slightly worse for RG, although no clear axis of colour discrimination loss was found. The number of daily smoked cigarettes seemed to be significantly related to the magnitude of discrimination loss, but only with the FM100h. Differences between moderate and heavy smokers were not significant in this sample. Error scores in smokers were positively correlated with the number of cigarettes smoked per day, and in BY also with age. Tobacco caused discrimination losses in both chromatic mechanisms but affected the red-green pathway more than the blue-yellow, and therefore, a partial RG score of FM100h test seems to be a good predictor of smoker colour deficiencies.

## Figures and Tables

**Table 1:** Data of 25, 50, and 75 percentiles by group for age, best-corrected visual acuity (*BCVA*), number of consumed cigarettes (CIGARS), FM100h scores (total scores (*TES*) and partial scores) for the red-green and blue-yellow mechanisms (*PTESRG, PTESBY*, respectively), colour axis index (*CA*), and gender (M: men; W: women).

	M/W	PERCENTILE	AGE	BCVA	CIGARS	*TES*	*PTESRG*	*PTESBY*	*CA*
**GROUP 0** ***n*** ** = 50**	31/19	25	32	1.16	0	32	9	22	0.21
		**50**	**42**	**1.38**	**0**	**56**	**19**	**32**	**1.47**
		75	54	1.50	0	75	30	47	2.43
**GROUP 1** ***n*** ** = 15**	4/11	25	28	1.16	4	56	21	34	0.00
		**50**	**35**	**1.20**	**8**	**76**	**34**	**45**	**1.14**
		75	51	1.46	10	128	52	72	1.50
**GROUP 2** ***n*** ** = 10**	2/8	25	28	1.12	20	90	34.3	54	0.39
		**50**	**35**	**1.32**	**20**	**128**	**49.5**	**71**	**1.55**
		75	51	1.50	40	194	87.3	106.8	1.93

Bold numbers represent the median data.

**Table 2:** Statistic results for the achromatic (A) and chromatic (RG and BY) contrast sensitivity comparison among groups at each frequency.

	1 Cpd		2 Cpd		4 Cpd		8 Cpd		16 Cpd	
	H	*p*-Value	H	*p*-Value	H	*p*-Value	H	*p*-Value	H	*p*-Value
**A**	4.214	0.122	0.1393	0.498	0.161	0.923	1.872	0.392	2.606	0.272
**RG**	6.022	0.049	3.258	0.196	3.336	0.189	0.0849	0.654	0.025	0.988
**BY**	0.615	0.735	2.334	0.311	3.236	0.198	0.322	0.851	0.249	0.883

**Table 3:** Constant, variable coefficients, and adjusted coefficient of determination for a linear regression model. Dependent variables: FM100h scores (*TES*, *PTESRG*, *PTESBY*, and computed principal component *C*); independent variables: age and number of smoked cigarettes.

	*TES*	*PTESRG*	*PTESBY*	*C*
**CONSTANT**	57.11	21.89	16.62	−0.35
**AGE Coeff.**	0	0	0.25	0
**CIGARETTES Coeff.**	3.90	1.88	0.30	0.07
**ADJUSTED R^2^**	0.44	0.41	0.41	0.44

**Table 4:** Extracted parameters from the ROC curves: area under the curves (AUC), sensitivity at 80% specificity, specificity at 80% sensitivity, and their respective cutoff values.

	AUC	Sensitivity	Specificity	Cutoff Value
***TES***	0.822	64%	80%	66
		80%	68	80
***PTESRG***	0.825	66%	80%	29.5
		80%	72%	32
***PTESBY***	0.788	60%	80%	35.3
		80%	50%	48
***C***	0.821	66%	80%	−0.23
		80%	70%	0.05
**CSF_RG_1**	0646	32%	80%	5.13
		80%	37%	3.88

## Data Availability

The data presented in the study are available on the request from the corresponding authors.
